# Copper-fixed quat: a hybrid nanoparticle for application as a locally systemic pesticide (LSP) to manage bacterial spot disease of tomato†

**DOI:** 10.1039/d0na00917b

**Published:** 2021-01-22

**Authors:** Ali Ozcan, Mikaeel Young, Briana Lee, Ying-Yu Liao, Susannah Da Silva, Dylan Godden, James Colee, Ziyang Huang, Hajeewaka C. Mendis, Maria G. N. Campos, Jeffrey B. Jones, Joshua H. Freeman, Mathews L. Paret, Laurene Tetard, Swadeshmukul Santra

**Affiliations:** Department of Chemistry, University of Central Florida Orlando FL 32826 USA ssantra@ucf.edu +1 407-882-2848; NanoScience Technology Center, University of Central Florida Orlando FL 32826 USA; Burnett School of Biomedical Sciences, University of Central Florida Orlando FL 32826 USA; Department of Physics, University of Central Florida Orlando FL 32826 USA; Department of Materials Science and Engineering, University of Central Florida Orlando FL 32826 USA; Plant Pathology Department, University of Florida Gainesville FL 32611 USA; North Florida Research and Education Center, University of Florida Quincy FL 32351 USA; Vocational School of Technical Sciences, Karamanoglu Mehmetbey University 70200 Karaman Turkey

## Abstract

The development of bacterial tolerance against pesticides poses a serious threat to the sustainability of food production. Widespread use of copper (Cu)-based products for plant disease management has led to the emergence of copper-tolerant pathogens such as *Xanthomonas perforans* (*X. perforans*) strains in Florida, which is very destructive to the tomato (*Solanum lycopersicum*) industry. In this study, we report a hybrid nanoparticle (NP)-based system, coined Locally Systemic Pesticide (LSP), which has been designed for improved efficacy compared to conventional Cu-based bactericides against Cu-tolerant *X. perforans*. The silica core–shell structure of LSP particles makes it possible to host ultra-small Cu NPs (<10 nm) and quaternary ammonium (Quat) molecules on the shell. The morphology, release of Cu and Quat, and subsequent *in vitro* antimicrobial properties were characterized for LSP NPs with core diameters from 50 to 600 nm. A concentration of 4 μg mL^−1^ (Cu): 1 μg mL^−1^ (Quat) was found to be sufficient to inhibit the growth of Cu-tolerant *X. perforans* compared to 100 μg mL^−1^ (metallic Cu) required with standard Kocide 3000. Wetting properties of LSP exhibited contact angles below 60°, which constitutes a significant improvement from the 90° and 85° observed with water and Kocide 3000, respectively. The design was also found to provide slow Cu release to the leaves upon water washes, and to mitigate the phytotoxicity of water-soluble Cu and Quat agents. With Cu and Quat bound to the LSP silica core–shell structure, no sign of phytotoxicity was observed even at 1000 μg mL^−1^ (Cu). In greenhouse and field experiments, LSP formulations significantly reduced the severity of bacterial spot disease compared to the water control. Overall, the study highlights the potential of using LSP particles as a candidate for managing tomato bacterial spot disease and beyond.

## Abstract 2

A hybrid core–shell silica nanoparticle system integrating Cu nanoclusters and Quat combats resistance development of *Xanthomonas perforans* responsible for bacterial spot disease of tomatoes.

## Graphical abstract

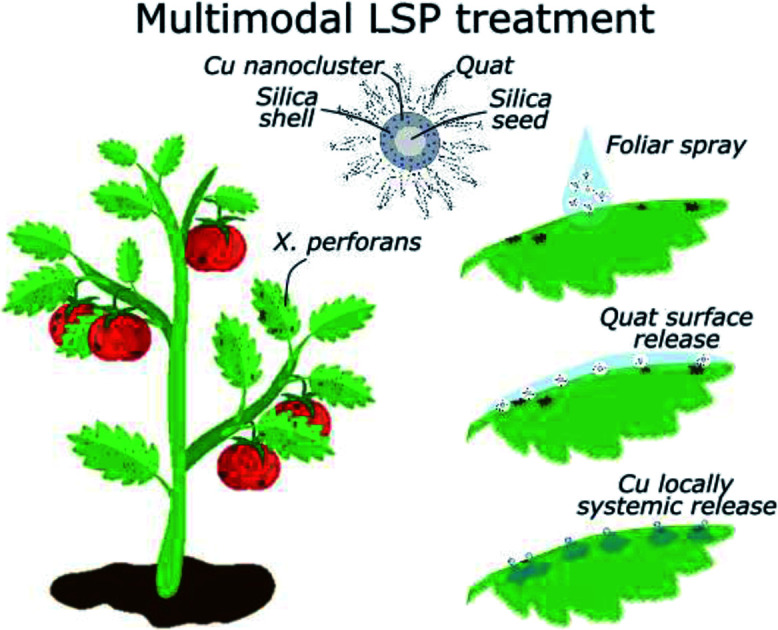

## Introduction

Tomato is one of the most important crops in the United States with production values of about $1.6 billion reported in 2019.^1^ Bacterial spot affects all above ground parts of the tomato plant including the foliage and fruits and has historically been controlled by using antibiotics such as streptomycin, which inhibits protein synthesis, and copper (Cu)-based bactericides, which damage the membrane through reactive radicals that can also damage biomolecules in the bacteria.^2–5^ With the reliance on streptomycin as the primary option for managing the disease in the 1950s, development of resistance occurred in a relatively short period of time and rendered the antibiotic ineffective. As streptomycin-resistant *Xanthomonas* strains emerged, Cu bactericides, offering a new mode of action, became widely used to manage bacterial spot disease.^6^ In turn, the extensive use of Cu bactericides has led to the development of Cu-tolerant bacterial strains in *Xanthomonas* spp. (*Xanthomonas gardneri*, *X. vesicatoria*, *X. euvesicatoria*, and *X. perforans*).^7^ Currently, Cu mixed with ethylene bis-dithiocarbamate-based materials, and containing other actives, such as manganese and zinc in mancozeb, is the standard treatment for bacterial spot.^8^ It has been discussed that copper–mancozeb offers higher bioavailability of Cu ions compared to Cu suspensions (cupric hydroxide).^7^ However, the lack of effective bactericides to potentially replace Cu-based formulations poses a real limitation to the tomato industry worldwide.^9–13^ Adding to this, heavy rainfalls, high temperatures, and moisture-rich climates create optimal conditions for transmission of the disease, making it increasingly difficult for growers to find effective management strategies.^5,8^ As a result, mixtures of multiple treatments of Cu at high concentrations are now used to maintain the production of tomatoes. Beyond the issue of development of Cu resistance in pathogens, extensive pesticide releases also impact the environment and public health.^14^

Limits in the amount of Cu sprayed are already being imposed by regulating agencies.^15^ Yet, achieving a sustainable replacement of Cu pesticides with safe alternatives constitutes a major obstacle. Engineered nanomaterials (ENMs) are expected to offer some benefits in food safety and production,^16^ however a lot of unknowns remain. Metals and metalloids of Ag,^17,18^ Ti,^19,20^ Mg,^21,22^ Zn,^23–26^ Mn^27,28^ have been studied as potential substitutes for Cu but the registration process of a new active is very cumbersome. In an effort to identify a short-term solution using the same Cu active ingredient, Strayer-Scherer *et al.* demonstrated that ultra-small size Cu nanoparticles (NPs) can provide comparable protection against Cu-tolerant strains of *X. perforans* at Cu concentrations five times lower than commercial formulations.^8^ To mitigate the phytotoxicity of Cu ions released from residues on plant leaves, fixed forms of Cu have been developed. For instance, silica gels loaded with ultra-small Cu NPs have demonstrated superior antimicrobial efficacy compared to conventional Cu bactericides.^29^ Loading Cu NPs in the silica matrix was found to offer minimal phytotoxicity at concentrations as high as 900 μg mL^−1^.^29^ Co-treatments, such as adding registered surfactants to ENMs containing Cu, are also being considered. For example, quaternary ammonium (Quat) compounds are positively charged ionic-surfactants commonly used as detergents, flocculants and disinfectants in commercial products.^30^ Quat compounds, such as didecyldimethylammonium chloride (DDAC), are membrane-active agents that impair the permeability of the bacterial membrane by binding to phospholipids and proteins. The use of Quat compounds in agriculture has been limited due to their high phytotoxicity.^31^ However, in a previous study, silica gel loaded with registered antimicrobial Quat, also referred to as “fixed-Quat”, provided antimicrobial efficacy and diminished phytotoxicity compared to free Quat molecules.^32^ Furthermore, fixed-Quat was shown to provide field efficacy against citrus canker comparable to that of Cu controls.^32^ Overall these new ENMs shed some light on potentially viable options to overcome bacterial resistance while reducing the amount of Cu released to the environment.

In this work, we designed a hybrid system containing Cu and Quat in a silica gel-like compound coined Locally Systemic Pesticide (LSP).^33^ By design (Fig. S1†), LSP particles contain a size tunable silica core (seed), a silica shell loaded with Cu NPs and a Quat compound. The synthesis of spherical LSP of different diameters was considered. We then evaluated the release of Cu and Quat from the formulation after washing with water to simulate rainfastness. The antimicrobial activity of the formulations was confirmed by minimum inhibitory concentration (MIC) assay. The interactions of the formulations with plant leaves were investigated and compared to water and conventional Cu pesticides such as Kocide 3000. Finally, the effects of the treatment on bacterial spot disease management in greenhouse and field conditions were evaluated.

## Methods

### Materials

All reagents were obtained from commercial vendors and used without further purification. These include: tetraethyl orthosilicate (TEOS, 98%, Gelest Inc., PA, USA), didecyldimethylammonium chloride (DDAC, 50% solution in 2-propanol/water 2 : 3 from EMD Millipore, MA, USA), ethanol (190 proof, Pharmco, CT, USA), ethanol (200 proof, Acros Organics, NJ, USA) and copper(ii) sulfate pentahydrate (99+%) (Acros Organics, NJ, USA), nitric acid (68 to 70% (w/w), Fisher Scientific, PA, USA), sodium hydroxide solid beads (Fisher Scientific, PA, USA), nutrient broth (NB) and agar (Fluka, St. Louis, MO, USA). *Xanthomonas alfalfae* subsp. *citrumelonis* (ATCC 49120), *Pseudomonas syringae* pv. *syringae* (ATCC 19310), and *Clavibacter michiganensis* subsp. *michiganensis* (ATCC 10202) cultures from ATCC (U.S. Department of Agriculture (USDA) permits P526P-12-04060 and P526P-15-01601) and *X. perforans* strains GEV485 (Cu-tolerant) and 91-118 (Cu-sensitive) isolated from tomato in Florida provided by Paret and Jones (North Florida Research and Education Center, University of Florida, USA) were used. Kocide 3000® (DuPont™) is a Cu-based fungicide/bactericide retaining 46.1% copper hydroxide (CAS No. 20427-59-2) available for commercial use for growers.

### Synthesis of LSP particles

LSP particles and corresponding controls were synthesized in one batch using sequential addition method. We previously reported the synthesis of silica gel loaded with ultra-small Cu NPs^29,34^ or Quat molecules.^32^ Similar methods were followed with minor revisions to achieve LSP particles combining both antimicrobial actives in a single particle delivery system. Three different sizes of LSP particles were synthesized from three different sizes of silica particles used as the LSP core. The three groups of particles will be labelled according to their final average size: LSP-50 nm, LSP-180 nm and LSP-600 nm.

A 200 mL stock LSP suspension with 10 000 μg mL^−1^ Cu and 2500 μg mL^−1^ Quat concentration was prepared. Silica seeds of three different sizes (seed #1, seed #2 and seed #3) were synthesized using the Stöber sol–gel method by changing respective ratios of reagents.^35,36^ Initially, 30% ammonium hydroxide_(aq)_ (2.8 mL for seed #1, 3.9 mL for seed #2, and 4.5 mL for seed #3) and deionized (DI) water (1.2 mL for seed #1, 4.5 mL for seed #2, and 11.25 mL for seed #3) were mixed in a 250 mL beaker, volumes adjusted to 50 mL with absolute ethanol. The beakers containing the mixtures were left on a magnetic stirrer at 400 rpm. In a separate graduated cylinder, 13.5 mL TEOS was added and the total volume adjusted to 150 mL using absolute ethanol. 50 mL of the prepared ethanol/TEOS mixture was then added into each beaker on the magnetic stirrer at a rate of 5 mL min^−1^. After completing the addition, the solutions were left to stir for 24 h. Seed #1 exhibited a core in the 30 to 45 nm range, seed #2 in the 120 to 150 nm range, and seed #3 in the 550 to 600 nm range based on DLS measurements (Fig. S2†).

The shell formation of LSP particles was achieved *via* hydrolysis of TEOS under acidic conditions. Initially, the pH of the suspensions of silica core (seed #1, #2, and #3) was lowered to 3 *via* addition of concentrated (50%) HNO_3(aq)_. In each suspension, 0.9 mL of TEOS was added dropwise. CuSO_4_·5H_2_O (7865 mg) was dissolved in 40 mL DI water, and then added to the solutions containing the silica seeds. After 4 h of mixing under magnetic stirring, the pH of the solutions was raised to 6.8 using 2 M NaOH(aq). Concentrated DDAC (1.11 mL) was added and the solutions were left to stir for 2 h. The final volume of all solutions was adjusted to 200 mL using DI water. Controls were synthesized by loading the particle shells with Cu only (LSP–Cu) in one case, and Quat only (LSP–Quat) in the other case. This was achieved by following the same procedure but replacing the second active by an equal amount of DI water. Representative images of solutions obtained from seed #1 for the formulation with two actives (LSP-50 nm) and for the controls with only one active (LSP–Cu-50 nm and LSP–Quat-50 nm) are provided in Fig. S3.†

### Characterization of LSP particles

#### Dynamic light scattering (DLS)

NP suspensions were prepared using 100 μL of stock and adding DI water to obtain a total volume of 10 mL before sonicating for 3 min immediately prior to the DLS measurement (He–Ne laser 633 nm, Zetasizer ZS90, Malvern Pananalytical, Malvern, UK). The pH of the solutions was adjusted to 6.8. After sonication, 1.2 mL of the suspension was placed in a clear disposable cuvette (DTS0012, Malvern Panalytical, Malvern, UK). The samples were diluted as necessary after the initial run to obtain count rates between 100 and 300 kcps while keeping the attenuator settings at 11 to obtain maximum laser power (4 mW).

#### Zeta potential measurements

Zetasizer (Malvern Zetasizer ZS90) was used to measure the surface charge of particles in all suspensions. Solutions were diluted using DI water and pH values were adjusted to 6.8. After dilution, 0.80 mL of prepared solution was pipetted into a disposable capillary cell (Malvern zeta folded capillary cell, DTS1070). The instrumental settings were adjusted to get a reading between 100–1000 kcps count rate, 0.1 to 10 mS cm^−1^ conductivity. Measurements were carried out in triplicates and average values of the surface charge were recorded.

#### Transmission electron microscopy (TEM)

25 μL of stock LSP solution was placed in a 15 mL volume glass vial and the final volume was adjusted to 10 mL with DI water. The diluted solution was sonicated for 30 min in an ultrasonic bath (Elmasonic S 30 H). 25 μL of the solution was drop-casted on the TEM grid (Electron Microscopy Sciences, CF300-AU-UL) and air-dried overnight. The measurements were carried out with a FEI Tecnai F30 TEM. Image analysis was carried out on the Gatan Microscopy Suite (GMS) 3 software.

#### Scanning electron microscopy (SEM)

Stock LSP solution (25 μL) was placed in a 15 mL volume glass vial and the final volume was adjusted to 10 mL with DI water. The diluted solution was sonicated for 30 min in an ultrasonic bath (Elmasonic S 30 H). 100 μL of the solution was drop-casted onto a freshly cleaned quartz microscope slide and air-dried overnight before being coated with gold by sputter coating (EMITECH) and imaged (ZEISS Ultra-55 FEG).

#### Raman spectroscopy

Raman spectra were collected using a confocal Raman system (WITec Alpha 300 RA) equipped with a 532 nm excitation laser with output power set at 16.6 mW, a 600 g mm^−1^ grating, and a CCD detector set at 10 s integration time. Spectra were baseline corrected using WITec Project. Noise reduction was applied with OriginLab 2017.

#### Release of Cu and Quat actives

The release of Cu and Quat from LSP particles was studied *in vitro*. Release of Cu studies were carried out on LSP-50 nm, LSP-180 nm, LSP-600 nm particles compared to reagent grade bulk cupric oxide, and cuprous oxide controls at 500 μg mL^−1^ concentration. 5 replicates for each sample were measured. 20 mL of 500 μg mL^−1^ LSP suspensions were prepared by diluting the prepared stock solution with DI water. The suspensions were then centrifuged at 11 000 rpm (Round Per Minute) for 5 min and the supernatants were separated for Cu and Quat quantification. After total removal of the supernatant, the precipitate was resuspended with 20 mL of DI water and centrifuged at 11 000 RCF for 5 min. This procedure was repeated 7 times. Cu content in the supernatant was measured by Atomic Absorption Spectroscopy (AAS) (Perkin Elmer Analyst 400). Quat concentration was quantified using the disulfine blue assay following a standard published protocol.^37^

### Characterization of wettability, simulated wash and phytotoxicity of LSP particles on leaves

#### Contact angle measurements

Citrus leaves were used as a model leaf for their relatively flat surface, to improve the consistency of the contact angle measurements. Young leaves were collected from a sweet orange tree and stored in a plastic bag with ice. Discs 2 cm in diameter were positioned on a glass slide with the leaf upper surface containing the waxy cuticle layer facing up. A 5 μL aliquot of LSP was dropped on the leaf surface from 5 cm above the surface. Pictures were acquired 30 s after the suspension drop reached the leaf using a camera located 25 cm away. The results were analysed using ImageJ. The tests were also carried out on tomato leaves (Fig. S4†) but contact angle values could not be interpreted due to the more complex leaf surface. Tomato leaves used were soft and hairy and did not provide a smooth enough surface for accurate contact angle calculations.

#### Simulated wash of LSP particles on leaves

The release of Cu upon wetting of LSP previously dried on the leaves was evaluated following the protocol described by Kah *et al.*^38^ with minor modifications. Newly emerged citrus leaves were collected, gently washed with DI water, and air dried. Plant surface areas were calculated using “Leafscan”, an iOS application. Leaves were sprayed with controls including DI water, Cu salt (Cu sulfate pentahydrate), Kocide 3000, LSP–Cu-50 nm, and with LSP-50 nm at 1700 μg mL^−1^ Cu concentration and 425 μg mL^−1^ Quat. Approximately 15 mL of solution were used to obtain completely saturated leaf surfaces. Following treatments, the leaves were left to air dry on a tray for 2 h.

For each simulated wash, a leaf was dipped into 30 mL DI water (pH 6.5) in a 50 mL centrifuge tube, which was gently tumbled for 30 s. The wash was repeated 3 times (wash 1 (W1), wash 2 (W2), wash 3 (W3)). After W3, surface bound Cu was removed by dipping the leaf into 30 mL dilute acid wash (AW) solution (2% HNO_3(aq)_ (v/v), 3% ethanol (v/v)) and tumbling for 1 min. The total metallic content in W1, W2, W3, and AW was analysed using a Cu standard calibration method obtained with AAS measurements. The percentage in Cu content after each wash was calculated using:
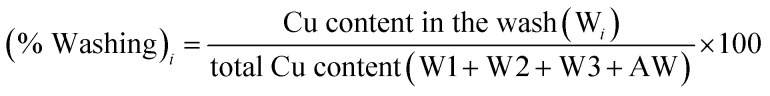


#### Phytotoxicity caused by LSP particles

Phytotoxicity was assessed on the model system, ornamental plant *Vinca* sp. (purchased from a local nursery). Previously established protocols demonstrated that *Vinca* plants revealed toxicity on leaves and flowers within 72 h of application due to free Cu and Quat.^32^ The same protocol to assess the phytotoxicity of LSP was followed. Plants were initially sprayed with DI water 24 h prior to the treatment and placed in a growth chamber (Panasonic MLR-352H) programmed to simulate summer conditions in the field (max. temperature 32 °C and humidity 85%). Controls included treating plants with DI water, Kocide 3000® at Cu concentrations of 1000 μg mL^−1^, DDAC (Quat) at concentration of 125 μg mL^−1^ and 250 μg mL^−1^, and Cu sulfate pentahydrate at Cu concentrations of 1000 μg mL^−1^. Applications of the LSP treatment at Cu concentrations of 500 μg mL^−1^ and 1000 μg mL^−1^ were performed using all-purpose spray bottles. Measurements were conducted in triplicate. Before 8 am (in growth chamber), the LSP suspensions were sprayed on the leaf surface until run-off. Phytotoxicity evaluation was carried out 72 h after treatment. The grading scheme used for assessment attributed: (−) non-phytotoxic (no brown spots), (+) mild phytotoxicity (a few visible scattered brown spots), (++) moderate phytotoxicity (multiple brown spots on multiple leaves), and (+++) severe phytotoxicity (large brown spots on most leaves).

### Characterization of bactericidal efficacy of LSP

#### Minimum inhibitory concentration (MIC) assay

MIC experiments were conducted using broth macro-dilution in accordance with the guidelines of the Clinical and Laboratory Standards Institute (CLSI).^39^ A range of concentrations from 0.5 μg mL^−1^ to 200 μg mL^−1^ active were tested for all LSP formulations and compared to controls LSP–Cu and LSP–Quat. Commercial controls included DDAC solution as the Quat source, and Kocide 3000 as commercial Cu standard. MIC was performed against Cu-tolerant *X. perforans* strain GEV 485, *P. syringae*, and *C. michiganensis* bacterial strains, grown and maintained at 28 °C. Each well contained ∼5 × 10^5^ CFU mL^−1^ of bacteria at the start of the 16–20 h incubation period.

#### Leaf simulation assay

Conditions simulating a leaf surface (*i.e.*, low nutrients) were used to determine the colony forming units (CFU). Cu-tolerant *X. perforans* GEV 485 was grown on nutrient agar (NA) plates containing 20 μg mL^−1^ Cu for 48 h at 28 °C. Bacterial suspensions were prepared in sterilized DI water amended with 0.01 M MgSO_4_ and adjusted to *A*_600_ = 0.3 (approximately 5 × 10^8^ CFU mL^−1^), then diluted to 10^5^ CFU mL^−1^. 20 μL of the bacterial suspensions were transferred to plate wells containing 2 mL of the following treatments (concentrations are indicated for each treatment): (1) 0.01 M MgSO_4_ for untreated control, (2) LSP-50 nm (5 μg mL^−1^ Cu : 1.25 μg mL^−1^ Quat, and 10 μg mL^−1^ Cu : 2.5 μg mL^−1^ Quat), (3) LSP-180 nm (5 μg mL^−1^ Cu : 1.25 μg mL^−1^ Quat, and 10 μg mL^−1^ Cu : 2.5 μg mL^−1^ Quat), (4) LSP-600 nm (5 μg mL^−1^ Cu : 1.25 μg mL^−1^ Quat, and 10 μg mL^−1^ Cu : 2.5 μg mL^−1^ Quat), (5) LSP–Quat-50 nm (1.25 μg mL^−1^ and 2.5 μg mL^−1^), (6) LSP–Quat-180 nm (1.25 μg mL^−1^ and 2.5 μg mL^−1^), (7) LSP–Quat-500 nm (1.25 μg mL^−1^ and 2.5 μg mL^−1^), (8) DDAC (Quat) (1.25 μg mL^−1^ and 2.5 μg mL^−1^), (9) LSP–Cu-50 nm (50 μg mL^−1^ and 100 μg mL^−1^), (10) LSP–Cu-180 nm (50 μg mL^−1^ and 100 μg mL^−1^), (11) LSP–Cu-500 nm (50 μg mL^−1^ and 100 μg mL^−1^), (12) cupric oxide CuO (50 μg mL^−1^ and 100 μg mL^−1^), (13) cuprous oxide Cu_2_O (50 μg mL^−1^ and 100 μg mL^−1^) and (14) Kocide 3000 (50 μg mL^−1^ and 100 μg mL^−1^).

The plates were incubated on a shaker (150 rpm) at 28 °C. Bacterial populations were measured by plating 50 μL from each well on NA after 1, 4 and 24 h of incubation followed by 48 h incubation at 28 °C.^8^ Colonies were counted to calculate the CFU per millilitre (CFU mL^−1^) for each time point. Measurements were performed in triplicate. The results were analysed using GraphPad Prism 7 with one-way ANOVA Dunnett test. Significant differences are reported using (*) for *p*-value < 0.05 when compared to the untreated control.

### Characterization of LSP efficacy in the greenhouse

LSP-50 nm, LSP-180 nm, and LSP-600 nm were tested against bacterial spot disease of tomato in one greenhouse trial. The following suspensions (200 mL each) were prepared in sterile tap water: LSP-50 nm, LSP-180 nm, LSP-600 nm, and Kocide® 3000 at 100, 200, and 500 μg mL^−1^. Sterile tap water served as the control. Approximately 30 mL of the material were sprayed on the foliage of 47 3- to 4-week-old Florida tomato plants. The leaf surfaces receiving the spray materials were allowed to air dry before spraying the leaf surfaces with a suspension of the Cu-tolerant *X. perforans* strain, GEV485, adjusted to 5 × 10^8^ CFU mL^−1^. The inoculated plants were then placed in plastic bags that were tightened around the base of the pot with a rubber band and placed in a growth chamber at 28 °C. After 48 h, the bags were removed and the plants transferred to the greenhouse. The plants were assessed for disease severity using the Horsfall–Barratt disease severity scale^40^ by rating every other day beginning 2 days post-inoculation, with the last rating 14 days post-inoculation. The disease rating assessed the overall affected area based on symptoms that included lesions, foliar blighting, and discoloration. The area under the disease progress curve (AUDPC) was then calculated using the midpoint values.^41^ The statistical significance of the data collected from the field experiment was evaluated using ANOVA followed by pair-wise comparisons with Least Significant Difference (LSD) in IBM® SPSS® Statistics Version 22. A *p*-value of <0.05 was used to evaluate significance.

### Characterization of LSP efficacy in the field

LSP-50 nm, LSP-180 nm, and LSP-600 nm were tested against bacterial spot disease of tomato in one field trial (8 July 2019 to 25 October 2019 in Quincy, FL). Four replicates consisting of 15 tomato plants (cv. Grand Marshall, Sakata Seed America, Fort Myers, FL) were carried out for each treatment. The plots were organized in an incomplete block design, and spaced 1.8 m apart with plants separated by 50.8 cm within the row.^42^ Tomato transplants were grown in 128-cell containers under greenhouse conditions before transplanting. Fertilizers applications followed cooperative extension recommendations.^43^ The treatments consisted of 100 and 500 μg mL^−1^ of LSP-50 nm, LSP-180 nm, and LSP-600 nm; Kocide 3000 (2.1 g L^−1^), Kocide 3000 (2.1 g L^−1^) in combination with Penncozeb 75DF (1.2 g L; United Phosphorus, Inc., King of Prussia, PA) (Cu–ethylene-bis-dithiocarbamate (EBDC)), and an untreated control (water). Every week until 1 week before fruit harvest, 1 L of each treatment was applied to each plot. Applications were done with a CO_2_ pressurized spray boom including 5 nozzles. From week 2 following field transplant of plants and treatment until harvest, bacterial spot disease severity and treatments phytotoxicity were assessed using the Horsfall–Barratt disease severity scale.^40^ The AUDPC was calculated after data collection.^41^ To assess the yield, 12 out of 15 plants were harvested. The 2 plants towards the two ends of the plots were excluded. 2 harvests are common for fresh market tomato production in Florida. Mature green or early breaker stage fruit were harvested and graded according to USDA standards.^44^ The statistical significance of the data collected from the field experiment was evaluated using ANOVA followed by pair-wise comparisons with Least Significant Difference (LSD) in IBM® SPSS® Statistics Version 22. A *p*-value of <0.05 was used to evaluate significance.

## Results and discussion

### Particle synthesis and characterization

It was previously reported that loading ultra-small Cu NPs to silica gels improves antimicrobial efficacy compared to conventional Cu bactericides, without any signs of phytotoxicity up to 900 μg mL^−1^ on *Vinca* plants.^29^ Similarly, Quat molecules can be loaded to silica gels and maintain their efficacy. Interestingly, this form of Quat, referred so as “fixed Quat”, significantly lowered the phytotoxicity observed in presence of free Quat molecules.^32^ Building upon these findings, LSP particles were designed to study the effect of combining fixed forms of Cu NPs and Quat on the ability to treat bacterial spot in tomato plants. The design uses silica NPs to host the two active ingredients, Cu and Quat (Fig. S1†). The hypothesis was that the incorporation of Cu NPs to the shell of the silica NPs would allow for high antimicrobial efficacy with slow Cu release to prolong efficacy after foliar application, which will be facilitated by the presence of Quat to increase membrane permeability. Furthermore, with Cu NPs embedded in the silica particles' shell, and fixed Quat linking the silica particles, the phytotoxicity associated with free Cu and Quat is expected to be lowered.

Three different silica core diameters were synthesized to compare the effects of size on the performance of the formulation. The first core diameter was in the nanoscale diameter range (LSP-50 nm) while the other two were above 100 nm (LSP-180 nm, and LSP-600 nm), which are comparable to conventional Cu pesticides.^45^ The Stöber sol–gel method was used to synthesize the silica seeds,^35^ as it allows for the synthesis of spherical, monodispersed particles ranging from nano- to micrometre size. The size of the particle seeds could be controlled by tuning the ratio of reactants to reaction media, or the temperature.^46^ Here the respective ratios of water, ammonia, and TEOS were varied while maintaining a total volume of 200 mL using ethanol. Consistent with previous reports, smaller size particles (∼50 nm) were successfully synthesized by lowering the water and ammonia content in the reaction media (see Materials and methods).^47^ The dimensions of the synthesized silica seeds were determined by DLS and electron microscopy (see Materials and methods). The hydrodynamic diameter was found to be 53 nm for seed #1, 180 nm for seed #2, and 500 nm for seed #3 (Fig. S2†). EM images further confirmed the seed diameters under vacuum state: 30–45 nm for seed #1, 120–150 nm for seed #2 and 550–600 nm for seed #3 (Fig. S5†).

**Fig. 1:**
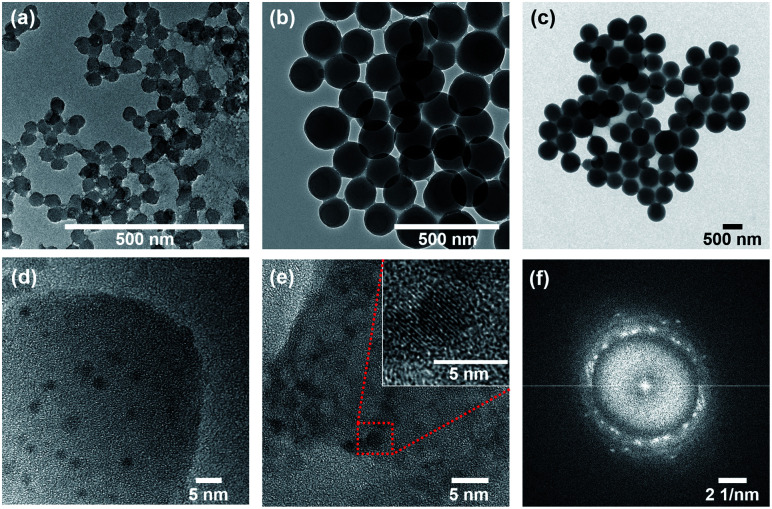
TEM images of (a) LSP-50 nm with particle size of 40–60 nm, (b) LSP-180 nm with particle size of 150–190 nm, and (c) LSP-600 nm with particle size of 570–650 nm. High resolution TEM images of (d, e) the Cu NPs embedded in the shell of LSP-50 nm particles and corresponding Fast Fourier Transform (FFT) data (f) indicating the crystalline nature of Cu NPs corresponding to copper hydroxide (JCPDS# 13-0420). The red box in (e) indicates one Cu NPs revealing crystalline orientation.

Next, the shells were synthesized, with incrusted Cu NPs and surface-bound Quat molecules (Fig. S1†). As a result, the hydrodynamic diameter increased from 53 to 74 nm for LSP-50 nm, from 180 to 220 nm for LSP-180 nm, and from 500 to 580 nm for LSP-600 nm (Fig. S2†). The increase in hydrodynamic size of LSP-50 nm (+21 nm) suggested that a shell of ∼10 nm was formed, while the thickness of the shell was ∼20 nm for LSP-180 nm and ∼40 nm for LSP-600 nm. Using TEM, the diameters were found to range between 40 to 60 nm for LSP-50 nm, 140 to 190 nm for LSP-180 nm, and 570 to 650 nm for LSP-600 nm, as shown in Fig. 1(a)–(c). SEM images (Fig. S6†) confirmed the size distributions and indicated the formation of a gel-like structure between the silica particles, likely due to hydrolysis of TEOS under acidic conditions.

Zeta potential measurements of the formulations (Fig. S9†) indicate that the surface charge of particles in Kocide 3000 (Cu) was negative (∼−38 mV). All LSP–Cu particles also exhibited negative surface charges. However, the addition of Quat to the cores resulted in surface charges either neutral or slightly positive. LSP particles with both Cu and Quat exhibited surface charges close to LSP–Quat for each size category. This can be understood from the interaction of Quat with the negatively charged silica gel surface. The surface charge of the particle is likely to influence the interaction of the formulation with the surface of the leaves. Avellan *et al.* reported that PVP-coated gold nanoparticles better interacted with leaf surfaces compared to citrate-coated gold nanoparticles due to their higher hydrophobicity.^52^ Hence, it is surmised that hydrophobic coatings will increase retention against rainfalls.

Composition of the gel was confirmed by Raman spectroscopy (Fig. S7†) and elemental analysis (Fig. S10†). The bands at 3100 cm^−1^ (LSP–Quat), 3140 cm^−1^ (LSP), and 3465 cm^−1^ (LSP and LSP–Quat), not observed in the fingerprint of LSP–Cu, confirm the presence of Quat in the formulation, which can be attributed to the gel linking the particles. The presence of Cu NPs, similar to nanoclusters embedded in the silica shell, was confirmed by TEM (Fig. 1(d) and (e)). Dark features in the silica shells with dimensions between 2.5 and 5 nm were analysed by two-dimensional Fast Fourier Transform (2D-FFT), which revealed 5 distinctive diffraction patterns (Fig. 1(f) and S8†) corresponding to *d*-spacing (lattice spacing) of 2.501 Å, 2.265 Å, 2.090 Å, 1.932 Å, and 1.780 Å consistent with copper hydroxide crystals (JCPDS# 13-0420) (Table S1†). This is in good agreement with previous report by Young *et al.*^29^

### Release of Cu and Quat actives

**Fig. 2:**
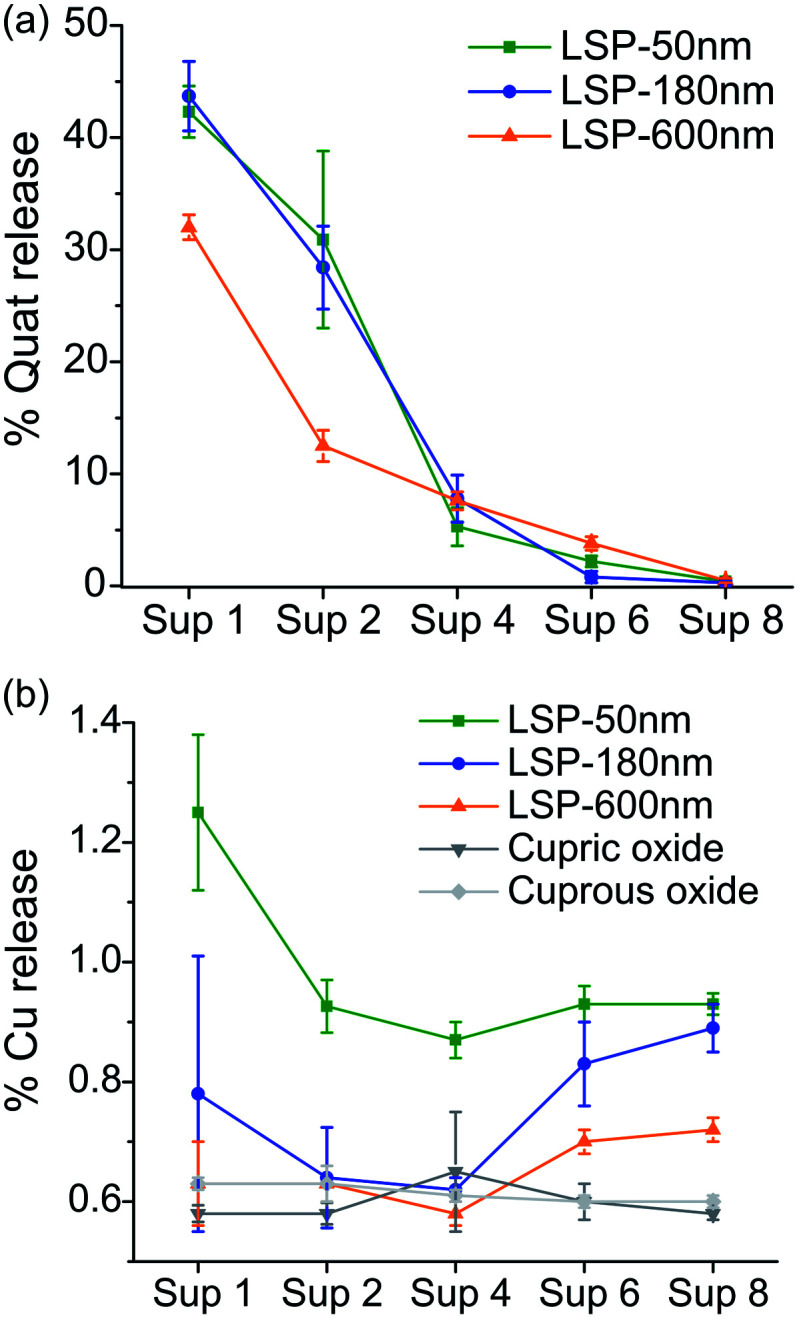
Percentage release of actives from LSP particles after washing with DI water, and separating supernatants (Sup) after each centrifugation. (a) Release of Quat molecule from LSP particles. (b) Release of Cu ions from LSP particles.

LSP particles were designed to slow down the release of actives. The quantification of Quat and Cu release from LSP is presented in Fig. 2(a) and (b), respectively (see Materials and methods). We inferred that with Quat molecules interacting electrostatically with the negatively charged silica surface, the molecules could be released under certain conditions such as at first application and first watering event (for instance with rain). The results indicate that the largest amount of Quat was released during the first (30–45%) and second (10–35%) washes, with more than 90% of Quat released for all particles by the 4^th^ wash. A significantly slower Quat release was observed during the first two washes for LSP-600 nm, suggesting that the size of the particles can affect the kinetics of release for such formulations.

On the other hand, embedding Cu NPs in the silica matrix was expected to slow the release of Cu ions to prolong the activity of the treatment. The release of Cu after each washing step for LSP particles is presented in Fig. 2(b), and compared to two Cu controls, cupric oxide and cuprous oxide. Only 0.6 to 1.3% of Cu was released during the first wash, which is significantly lower than the 30–45% Quat loss (Fig. 2(a)). LSP-50 nm demonstrated the highest release of Cu for the 8 washes, indicating that the size of the silica particle and the shell can be used to control the delivery of actives. LSP-180 nm and LSP-600 nm exhibited comparably low (below 1%) release during the first 4 washes but observed an increase for washes 5 to 8. This suggests that the removal of Quat at the surface of the particles can affect the rate at which Cu becomes bioavailable. The analysis of the supernatant with DLS confirmed no detectable NPs (data not shown), suggesting that Cu is released mostly in its ionic form.

The weak electrostatic interaction of Quat molecules with the negatively charged silica surface facilitates their release after a few washes, which is beneficial for rapid antibacterial action on the leaves upon foliar application. Cu release from the silica shell, which seems to be in the form of Cu ions given the absence of signal from particulate matter in DLS measurements, could provide antibacterial efficacy over time, although the concentration released should be adjusted to ensure killing of target pathogens (*i.e.*, above MIC level).

### Characterization of bactericidal efficacy of LSP

MIC and leaf simulation assays were carried out to quantify the efficacy of LSP particles (see Materials and methods). MICs were evaluated against Cu-tolerant *X. perforans* (GEV 485), *Pseudomonas syringae* pv. *syringae* (ATCC 19310), and *Clavibacter michiganensis* subsp. *michiganensis* (ATCC 10202) (Table 1). LSP-50 nm, LSP-180 nm, and LSP-600 nm particles achieved high efficacy against the three bacterial strains. MIC of 4 μg mL^−1^ (Cu) : 1 μg mL^−1^ (Quat) was obtained against *X. perforans* and *P. syringae* pv. *syringae* and MIC of 2 μg mL^−1^ (Cu) : 0.5 μg mL^−1^ (Quat) when treating *C. michiganensis* subsp. *michiganensis*. These MICs constitute a significant improvement from the MIC of 100 μg mL^−1^ and 200 μg mL^−1^ observed for Kocide 3000 (metallic Cu). Leaving Quat out of the formulations (*i.e.*, LSP–Cu) negatively affected the MIC, although the treatment exhibited a single dilution improvement compared to Kocide 3000, thus validating the benefit of using Cu NPs to improve antibacterial efficacy. Removing Cu from the formulations (*i.e.*, LSP–Quat) did not affect the MIC. However, from the release study (Fig. 2), we surmise that the plant protection with LSP–Quat would be short-lived in the field, as Quat would be washed off after a few rainy events. No difference on MIC values could be detected between LSP-50 nm, LSP-180 nm, and LSP-600 nm in the conditions tested.

**Table 1:** Minimum Inhibitory Concentration (MIC) of LSP treatments tested against Cu-tolerant *X. perforans*, and *P. syringae*, and *C. michiganensis* bacterial strains

MIC (μg mL^−1^)			
	*X. perforans* (GEV 485)	*Pseudomonas syringae* pv. *syringae* (ATCC 19310)	*Clavibacter michiganensis* subsp. *michiganensis* (ATCC 10202)
LSP-50 nm	Cu: 4	Cu: 4	Cu: 4
	Quat: 1	Quat: 1	Quat: 0.5
LSP-180 nm	Cu: 4	Cu: 4	Cu: 4
	Quat: 1	Quat: 1	Quat: 0.5
LSP-600 nm	Cu: 4	Cu: 4	Cu: 4
	Quat: 1	Quat: 1	Quat: 0.5
Quat (DDAC)	1	2	0.5
LSP–Quat-50 nm	1	2	0.5
LSP–Quat-180 nm	1	2	0.5
LSP–Quat-600 nm	1	2	0.5
LSP–Cu-50 nm	50	50	100
LSP–Cu-180 nm	50	50	100
LSP–Cu-600 nm	50	50	100
Kocide 3000	100	100	200

**Fig. 3:**
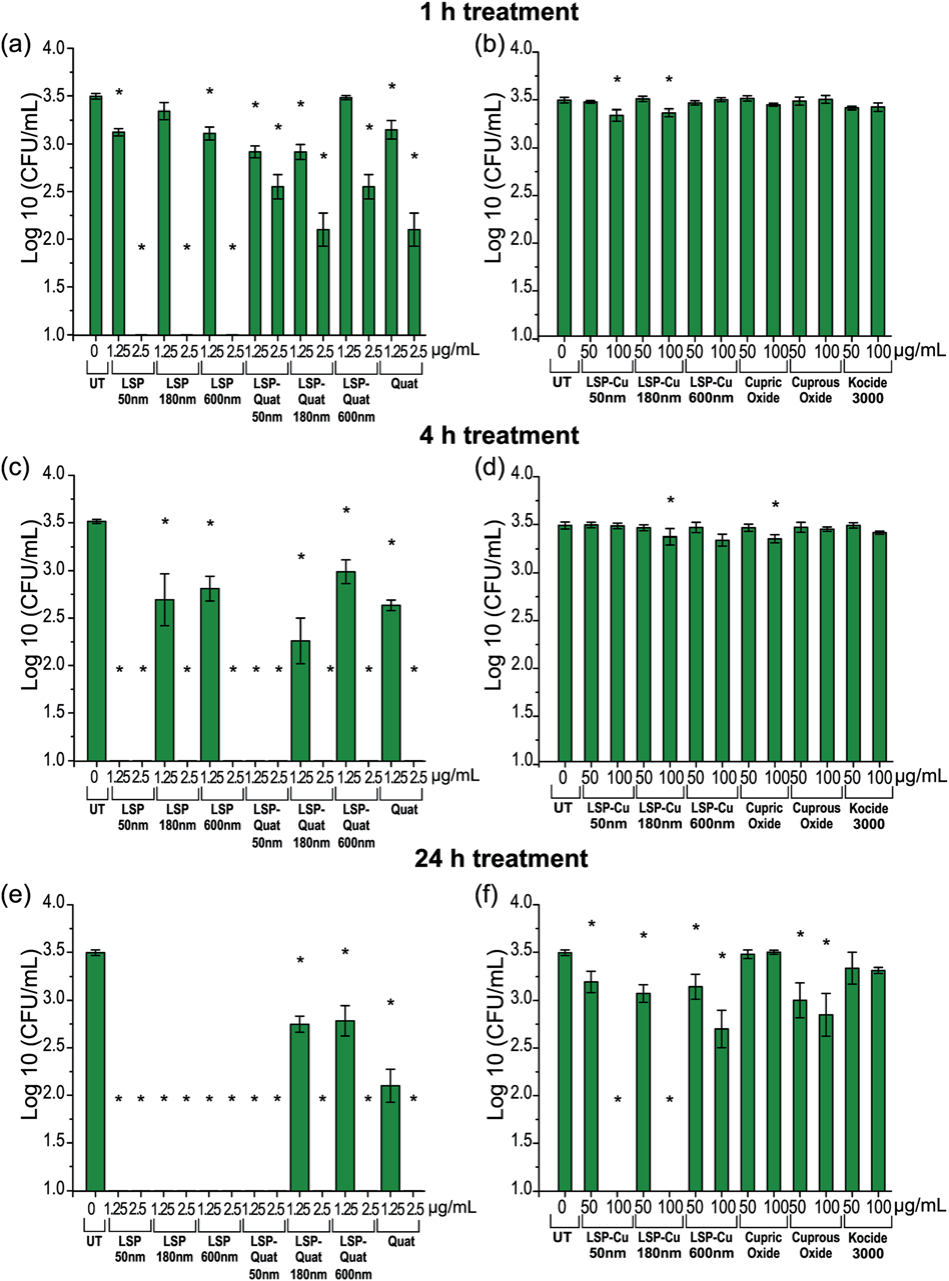
*X. perforans* GEV 485 viability at different time points after exposure to Cu and Quat in LSP, LSP–Quat, LSP–Cu and controls (a and b) after 1 h of treatment, (c and d) after 4 h of treatment and (e and f) after 24 h of treatment. Analysis with one-way ANOVA Dunnett was used to identify significant differences reported using a (*) with a *p*-value < 0.05 compared to the untreated control.

The viability of Cu-tolerant *X. perforans* following exposure to LSP was further investigated by determining CFU in low nutrient conditions to simulate the environment on the leaf surface where bacteria land during rain events (see Materials and methods). Bacteria treated with LSP, LSP–Quat, and DDAC (Quat) were analysed after 1 h (Fig. 3(a)), 4 h (Fig. 3(c)), and 24 h (Fig. 3(e)). Bacteria treated with LSP–Cu, cupric oxide, cuprous oxide, and Kocide 3000 were also assessed after 1 h (Fig. 3(b)), 4 h (Fig. 3(d)), and 24 h (Fig. 3(f)). Treatments with 2.5 μg mL^−1^ of Quat for 1 h lead to a significant decrease in bacteria viability, especially in the case of LSP. LSP–Quat-50 nm inhibited the growth of bacteria at 1.25 μg mL^−1^ at 4 h and 24 h, while the larger LSP–Quat-180 nm and LSP–Quat-600 nm required a higher concentration of 2.5 μg mL^−1^, even at 24 h (Fig. 3(e) and (f)). DDAC (Quat) also required a concentration of 2.5 μg mL^−1^ to observe growth inhibition at 24 h. Kocide 3000 did not exhibit any killing efficacy against Cu-tolerant *X. perforans*, even after 24 h exposure to a concentration of 100 μg mL^−1^. Comparatively, treatment with LSP–Cu-50 nm and LSP–Cu-180 nm led to decrease in colonies at Cu concentration of 50 μg mL^−1^ after 24 h. LSP–Cu treatments at 100 μg mL^−1^ showed a significant effect at 1 h and completely killed off colonies at 24 h. An effect of LSP particle size was observed with the LSP–Cu formulations, where LSP–Cu-50 nm and LSP–Cu-180 nm completely killed the bacteria after 24 h at 100 μg mL^−1^, whereas a reduced population remained at that concentration LSP–Cu-600 nm (Fig. 3(f)). Overall, MIC and CFU assays confirmed the enhanced antimicrobial effect of LSP with two actives.

### Characterization of the wettability, simulated wash and phytotoxicity of LSP particles

To analyse wettability of the formulations, we deposited a drop of solution on the surface leaves and analysed the contact angle (see Materials and methods). Leaf surfaces are water repelling due to their hydrophobic cuticle and hairy surfaces.^48^ When agrochemical formulations are foliar sprayed in the field, droplets usually exhibit poor wettability properties, as seen from the high contact angle of the Kocide 3000 droplet with the leaf surface in Fig. S4.† High contact angles correspond to minimal interaction with the leaf, resulting in droplets beading up and rolling off the leaf.^49^ This is known to limit treatment efficacy. A common remedy to this has been to increase the quantity of agrochemical used, resulting in high quantity of treatment reaching the soil. Surfactants, a type of adjuvant, are often mixed with formulations to reduce surface tension and contact angles of droplets on leaves.^50,51^ In LSP, the use of Quat was found to significantly increase surface wetting of LSP treatments. To quantify this, young citrus leaves, ideal for their consistently flat surfaces, were used. Fig. S4† summarizes the contact angle measurements obtained when depositing LSP, LSP–Cu and LSP–Quat. The results are compared to contact angles measured for DI water and Kocide 3000. We note that the lower the angle, the better the wettability of the product. Angles above 90° suggest hydrophobicity and correspond to a drop that can easily roll off the surface. Overall, the drops from the LSP–Quat formulations demonstrated the lowest contact angle (37–40.9°), corresponding to the best surface wetting. All LSP formulations containing Quat demonstrated better surface wetting than water (93.7°) and Kocide 3000 (85.2°), known as a film forming product. LSP–Cu formulations exhibited contact angles similar to Kocide 3000, between 75.8° and 86.8°. LSP treatments containing Quat offer a means to wet leaf surfaces better than existing film-forming products. Hence Quat in LSP is expected to improve the effect of the treatment in field applications by improving wetting as well as by boosting the antibacterial efficacy due to its chemical action at the leaf surface upon application. Surface wetting was also studied on tomato leaves, although the complexity of their surface only allowed for qualitative comparison of the treatments (Fig. S4†). Similarly, to what was observed on citrus leaves, formulations containing Quat demonstrated superior surface wetting.

**Fig. 4:**
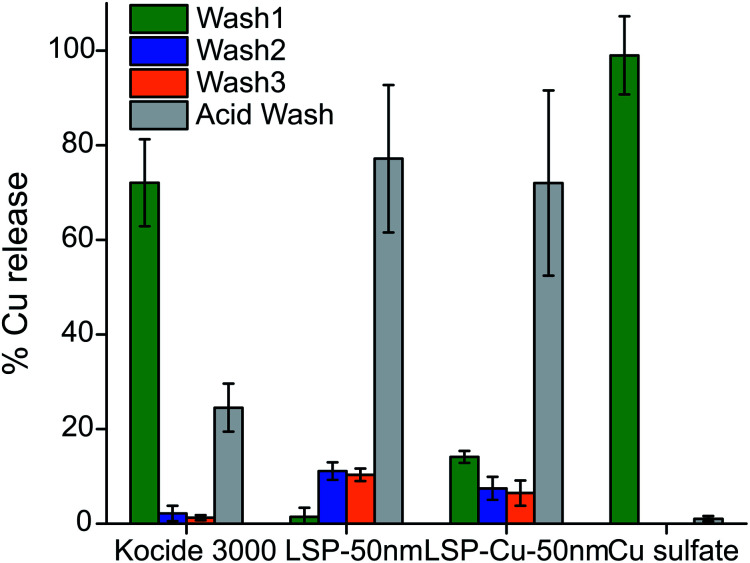
AAS analysis of the Cu content released after washing-off LSP particles from leaf surfaces with 3 washes with DI water and a diluted acid wash.

To further investigate the extent to which antibacterial agents persist on the leaves after rainfalls, we conducted wash-off simulations (see Materials and methods) and determined the amount of Cu released from leaves treated with LSP-50 nm, LSP–Cu-50 nm, Cu sulfate, and Kocide 3000. The quantity of Cu released was measured by AAS (Fig. 4, see Materials and methods). After letting the treatments dry on the leaves, 3 cycles of water washing were performed with DI water, followed by one acid wash (AW) to remove the remaining Cu.

**Fig. 5:**
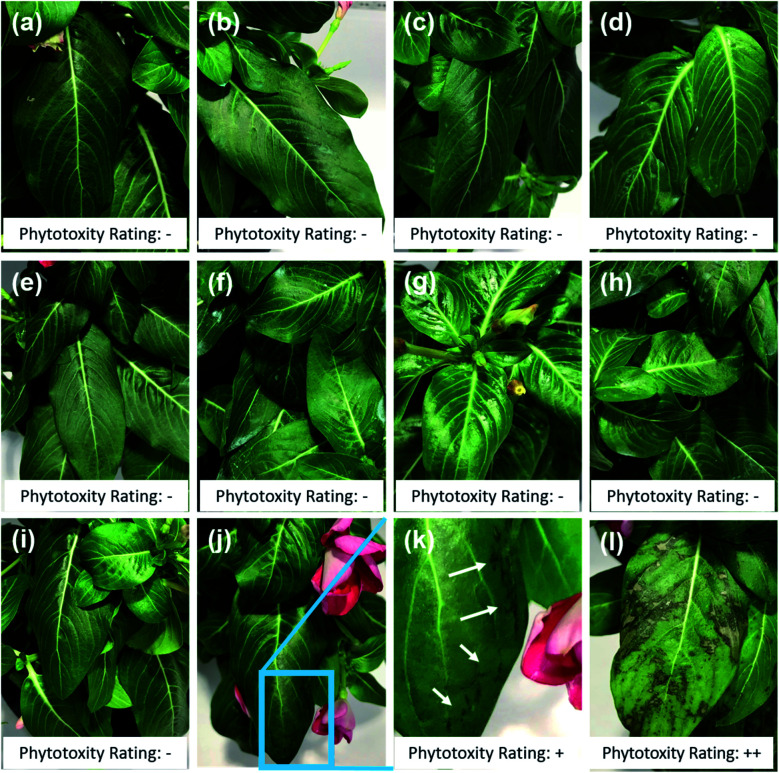
Phytotoxicity assessment of LSP particles on *Vinca*. Representative images acquired after 72 h of foliar application of (a) DI water, (b) Kocide 3000 at Cu concentration of 1000 μg mL^−1^, (c) LSP-50 nm at 500 μg mL^−1^ and (d) 1000 μg mL^−1^, (e) LSP-180 nm at 500 μg mL^−1^ and (f) 1000 μg mL^−1^, (g) LSP-600 nm at 500 μg mL^−1^ and (h) 1000 μg mL^−1^, (i) Quat at 125 μg mL^−1^, (j and k) Quat 250 μg mL^−1^ where (k) is a zoomed in view of the leaf exhibiting signs of phytotoxicity, (l) copper sulfate at 1000 μg mL^−1^. Phytotoxicity rating: − indicates no toxicity, + low toxicity, +++ severe toxicity.

Close to 95% of the Cu content was removed from the leaves during the first water wash in the case of leaves treated with Cu sulfate, which is water soluble. In the case of leaves treated with Kocide 3000, more than 60% of Cu content was released during the first wash, and 36% during the AW. The leaves treated with LSP-50 nm and LSP–Cu-50 nm retained more than 90% of Cu after W1, and more than 70% after the W3 but released 3 times more Cu in the AW than the leaves treated with commercial products. This suggests that as long as the gel-like LSP films remain on the leaves, Cu residues are available for release on the foliage of the plants. The results are in agreement with a study by Kah *et al.*, which reported that nano-formulations help increase the amount of Cu available and the duration for which Cu is available (in number of washes) compared to Cu from bulk compounds.^38^ In turn, as treatments remain longer on the plants, it is important to assess their phytotoxicity. In previous studies, *Vinca* spp. plants were found to reveal phytotoxicity within 72 h of foliar application, with higher sensitivity than tomato plants.^32^ Following the same protocol, we carried out a comparative visual assessment of phytotoxicity of LSP treatments on *Vinca* spp. 72 h after foliar application (Fig. 5). Water-soluble Cu sulfate sprayed at 1000 μg mL^−1^ caused severe phytotoxicity in the form of large brown spots (Fig. 5(l)) while plants treated with DI water and Kocide 3000 did not display any sign of phytotoxicity (Fig. 5(a) and (b)).

DDAC (free Quat) did not show any sign of phytotoxicity at concentration of 125 μg mL^−1^ but several lesions at the edge of the leaves were observed at 250 μg mL^−1^ (Fig. 5(j) and (k)). All three sizes of LSP particles were found to be non-phytotoxic at both Cu concentrations of 500 μg mL^−1^ and 1000 μg mL^−1^ (Fig. 5(c)–(h)). This constitutes an improvement compared to previously reported fixed-Quat gels, which exhibited phytotoxicity on *Vinca* spp. at concentration of 1000 μg mL^−1^. Overall, the results suggest that the known phytotoxicity of soluble Quat and Cu can be mitigated by the gel-like arrangement of core–shell LSP particles.

### Characterization of LSP efficacy in the field

**Fig. 6:**
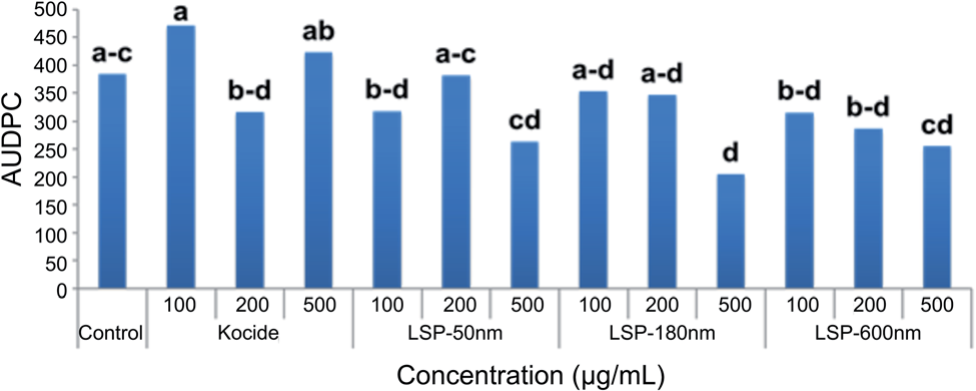
Efficacy of LSP particles tested under greenhouse settings. Field efficacy of LSP particles compared to growers standard Cu product.

Greenhouse efficacy of LSP-50 nm, LSP-180 nm, and LSP-600 nm were assessed against Cu-tolerant *X. perforans* (GEV 485), the most dominant species found in Florida tomato fields. Fig. 6 represents the disease control level achieved with LSP treatments compared to sterile water and Kocide 3000. The disease severity was assessed every 2 days for 14 days (see Materials and methods). The calculated AUDPC indicates that LSP-50 nm and LSP-600 nm at 100 μg mL^−1^ (Cu concentration) affected the leaves at a level similar to Kocide 3000 applied at 200 μg mL^−1^. LSP-50 nm and LSP-600 nm at 500 μg mL^−1^ achieved inhibition of disease progression, which was slightly surpassed by LSP-180 nm at the same concentration. We note that Kocide 3000, even at 500 μg mL^−1^, did not exhibit any strong sign of disease control in comparison to sterile water control. Following greenhouse testing, one field experiment was conducted in Fall 2019 at Quincy, FL (Table 2). At the concentration of 500 μg mL^−1^, both LSP-180 nm and LSP-600 nm significantly reduced disease severity compared to the untreated control group, whereas the grower's standard Cu–EBDC (Penncozeb 75DF) did not significantly reduce disease in the field trials compared to the untreated control (*p* < 0.05) (Table 2). In addition, Kocide 3000 treated plots had significantly higher disease severity compared to the untreated control (*p* < 0.05). Even though LSP-50 nm had slightly better *in vitro* antimicrobial efficacy, the two other LSP particles performed better under field conditions. Based on the field results, LSP-180 nm and LSP-600 nm have the potential to be an alternative to traditional Cu formulations. No phytotoxicity on the tomato plants was observed on plants treated with LSP (data not shown).

**Table 2:** Comparison of the efficacy of LSP (LSP-50 nm, LSP-180 nm, and LSP-600 nm), copper (Kocide 3000), and grower standard (Cu–EBDC) to manage bacterial spot disease severity (area under disease progress curve – AUDPC) on tomato variety ‘Grand Marshall’ in a field experiment in Quincy, FL

Treatments	Rate (μg mL^−1^)	AUDPC^b^
LSP-50 nm	100	2095.4 *ab*^c^
LSP-50 nm	500	1869.0 *ab*
LSP-180 nm	100	2053.6 *ab*
LSP-180 nm	500	1769.6 *a*
LSP-600 nm	100	2001.4 *ab*
LSP-600 nm	500	1774.9 *a*
Kocide 3000		2636.1 *c*
Cu–EBDC^a^		1974.8 *ab*
Water		2188.2 *b*

a Cu–EBDC is composed of Kocide 3000 (2100 μg mL^−1^) and Penncozeb® 75DF (1200 μg mL^−1^).

b The area under disease progress curve (AUDPC) was calculated using the midpoint values of Horsfall–Barratt disease severity scale.

c Number with different character in the same column has significant difference (*p*-value = 0.05) based on Least Significant Difference statistical analysis using the IBM® SPSS® program.

However, no significant impact on total marketable yield and extra-large fruit yield was observed as a result of LSP treatments in the field trial (Table 3). It should be noted that in the greenhouse experiment, test compounds were only applied one time, which could be a factor for only a margin disease management activity, with only LSP-180 nm showing significant reduction in disease severity compared to the control. On the other hand, in the field experiment the test compounds were applied several times, which lead to significant effects in reducing disease severity by both LSP-180 nm and LSP-600 nm at the highest concentration compared to the water and copper control, and LSP-50 nm at the highest concentration compared to copper control.

**Table 3:** Total marketable yield (kg ha^−1^) and extra-large (X-Large) fruit (kg ha^−1^) in the field experiment following treatment of tomato plants ‘Grand Marshall’ with LSP (LSP-50 nm, LSP-180 nm, and LSP-600 nm), Cu (Kocide 3000), and the grower standard (Cu–EBDC) in Quincy, FL

Treatments	Rate (μg mL^−1^)	Total yield			X-Large fruits		
		Average	SE^a^	LSD	Average	SE	LSD
LSP-50 nm	100	52 688.78	6347.11	*a* c	13.08	2.40	*a*
LSP-50 nm	500	53 377.37	3132.43	*a*	15.94	1.97	*a*
LSP-180 nm	100	63 573.05	8115.07	*a*	16.72	2.31	*a*
LSP-180 nm	500	71 236.47	9250.87	*a*	20.66	4.14	*a*
LSP-600 nm	100	63 039.94	6835.63	*a*	18.41	4.27	*a*
LSP-600 nm	500	52 488.86	7170.98	*a*	14.74	2.66	*a*
Kocide 3000		67 260.38	6617.58	*a*	17.84	1.84	*a*
Cu–EBDC^b^		65 039.10	5049.82	*a*	20.80	3.53	*a*
Water		65 955.38	12 031.39	*a*	19.74	4.38	*a*

a SE is Standard Error.

b Cu–EBDC is composed of Kocide 3000 (2100 μg mL^−1^) and Penncozeb® 75DF (1200 μg mL^−1^).

c Number with different character in the same column has significant difference (*p*-value = 0.05) based on Least Significant Difference (LSD) statistical analysis using the IBM® SPSS® program.

## Conclusion

In summary, although additional field studies would need to be undertaken to optimize disease management conditions, the ability to tune the size and concentration of Cu and Quat loaded onto the silica core of the LSP particle has the potential to provide a new nanobiocide platform to overcome developing resistance in bacteria responsible for bacterial spot disease in tomatoes. The hydrophobicity of the LSP formulations, observed from the zeta potential measurements, are likely to increase the retention on the leaves during rainfalls. In fact, LSP formulations exhibited better surface wetting and longer retention of Cu on the leaves than Kocide 3000 and Cu sulfate, but no phytotoxicity despite the presence of Quat. The concept could in turn be applied to target other pathogens given that MIC and CFU values indicate efficacy on various strains. The significant reduction in phytotoxicity of otherwise highly reactive agents such as free Cu ions and free Quat molecules also paves the way toward material by design for a more effective agriculture.

## Author contributions

AO synthesized the nanoparticles at UCF. AO, ZH and MGNC performed the material characterization and phytotoxicity studies. BL carries out the Raman spectroscopy measurements. MY carried out the bacterial work. HCM contributed to designing and carrying out phytotoxicity studies with AO and MGNC. YYL, SDS, DG, JC and JHF carried out the greenhouse and field trials. AO, BL, JBJ, MLP, LT and SS wrote the manuscript. All authors contributed to the manuscript revisions, AO did so from his current institution.

## Conflicts of interest

There are no conflicts to declare.

## Supplementary Material

NA-003-D0NA00917B-s001
