# Supplementary material for: Copper-fixed quat: a hybrid nanoparticle for application as a locally systemic pesticide (LSP) to manage bacterial spot disease of tomato
Source: Nanoscale Adv. 2021 Jan 22;3(5):1473–83. doi: 10.1039/d0na00917b (PMC9417342; doi:10.1039/d0na00917b)
Supplement: NA-003-D0NA00917B-s001 [file NA-003-D0NA00917B-s001.pdf]

Electronic Supplementary Material (ESI) for Nanoscale Advances.
This journal is © The Royal Society of Chemistry 2021

Supporting information

Copper-fixed quat: A hybrid nanoparticle for application as a Locally Systemic Pesticide (LSP) to manage bacterial spot disease of tomato

Ali Ozcan[1, 2,ǂ], Mikaeel Young[2,3], Briana Lee[2], Ying-Yu Liao[6,7], Susannah Da Silva[6], Dylan Godden[6], James Colee[6], Ziyang Huang[1,2], Hajeewaka C. Mendis[2], Maria G. N. Campos[2], Jeffrey B. Jones[6], Joshua H. Freeman[6], Mathews L. Paret[6,7], Laurene Tetard[2,4], Swadeshmukul Santra[1,2,3,5 *]

[1]Department of Chemistry, [2]NanoScience Technology Center, [3]Burnett School of Biomedical Sciences, [4]Department of Physics and [5]Department of Materials Science and Engineering, University of Central Florida, 4353 Scorpius St., Suite 245, Orlando, FL 32826, USA
[6] Plant Pathology Department, University of Florida, Gainesville, FL 32611, USA
[7] North Florida Research and Education Center, University of Florida, Quincy, FL, 32351, USA
[ǂ] Current address: Vocational School of Technical Sciences, Karamanoglu Mehmetbey University,70200, Karaman, Turkey
*Phone: 407-882-2848; Fax: 407-882-2819; E-mail: ssantra@ucf.edu

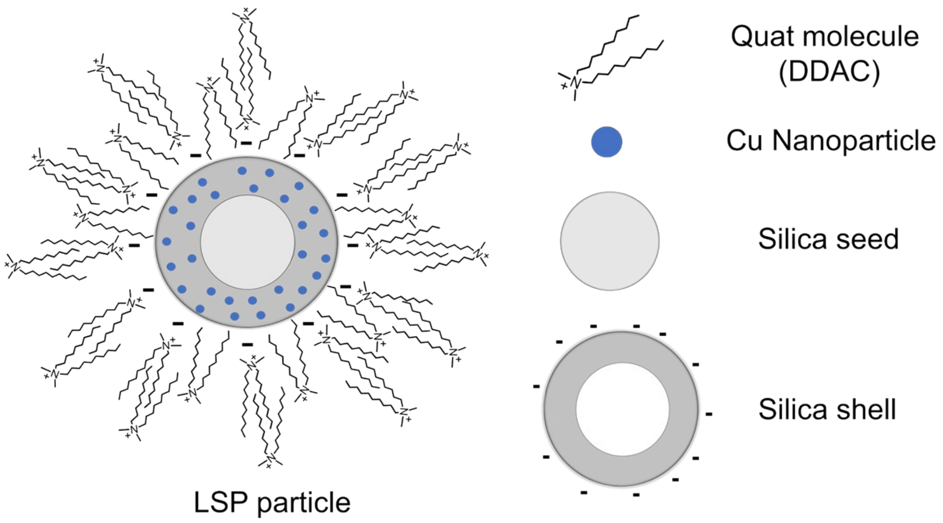

Figure S1: Schematics of LSP particle design. The inert silica seed reduces the amount of copper necessary to maintain antimicrobial efficacy, which is boosted by incorporated nanoclusters of copper in the shell. A second antibacterial agent, Quat, is also bound to the shell to increase the modes of action and efficacy against bacterial diseases.

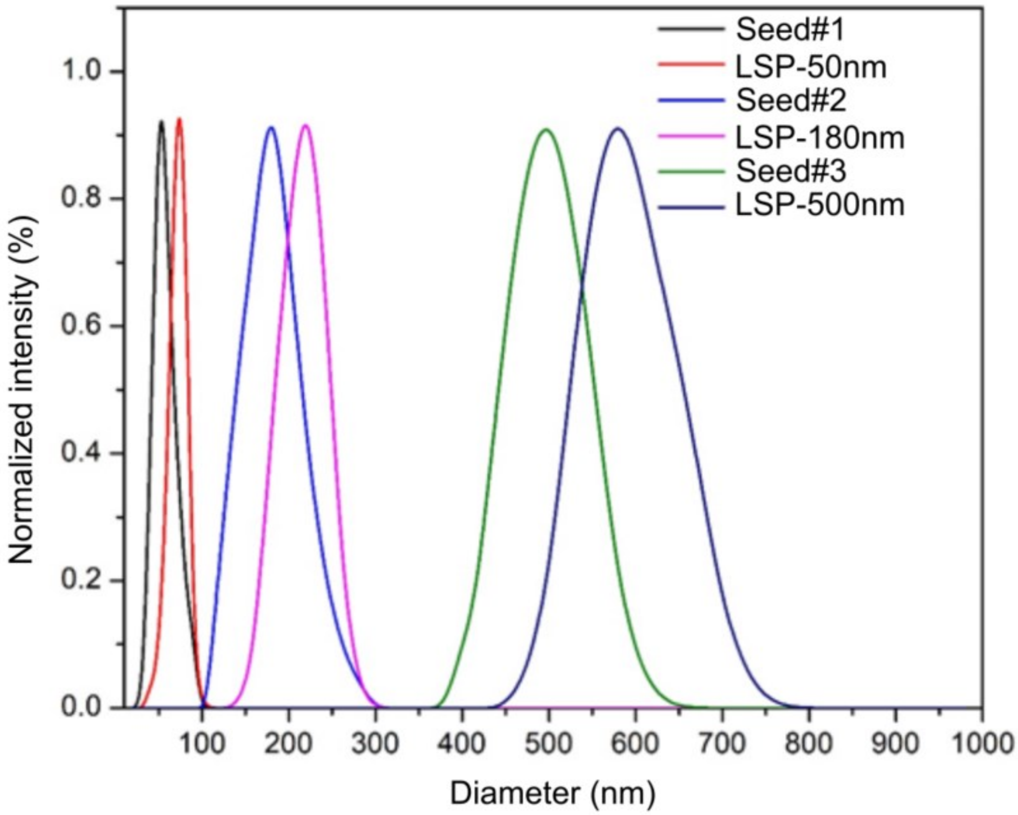

Figure S2: Hydrodynamic size distribution of silica seeds (seed#1, seed#2, seed#3) and resulting LSP particles after adding the shell with 2 actives (LSP-50nm, LSP-180nm, LSP-600nm) determined by DLS.

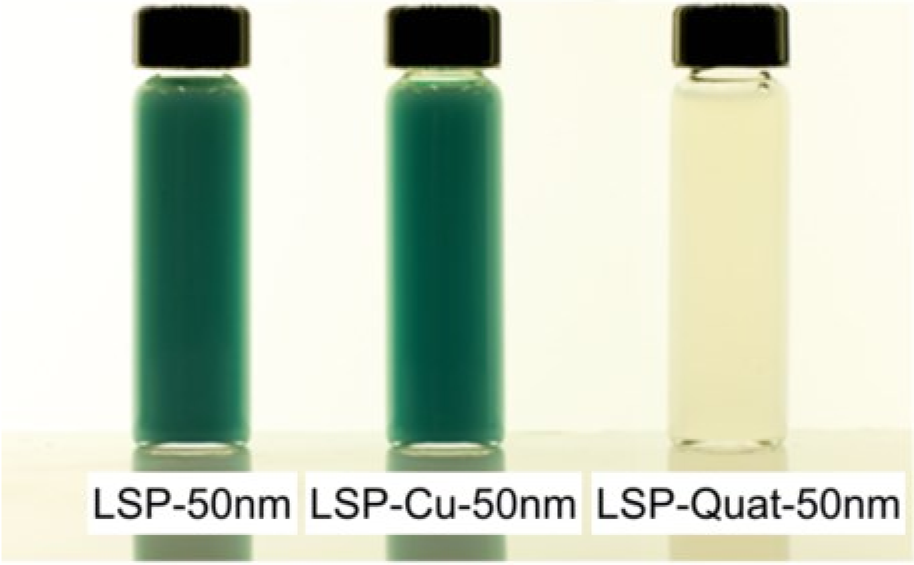

Figure S3: Solution of LSP-50nm (left), control with Cu-active only LSP-Cu-50nm (center) and control with Quat only LSP-Quat-50nm (right).

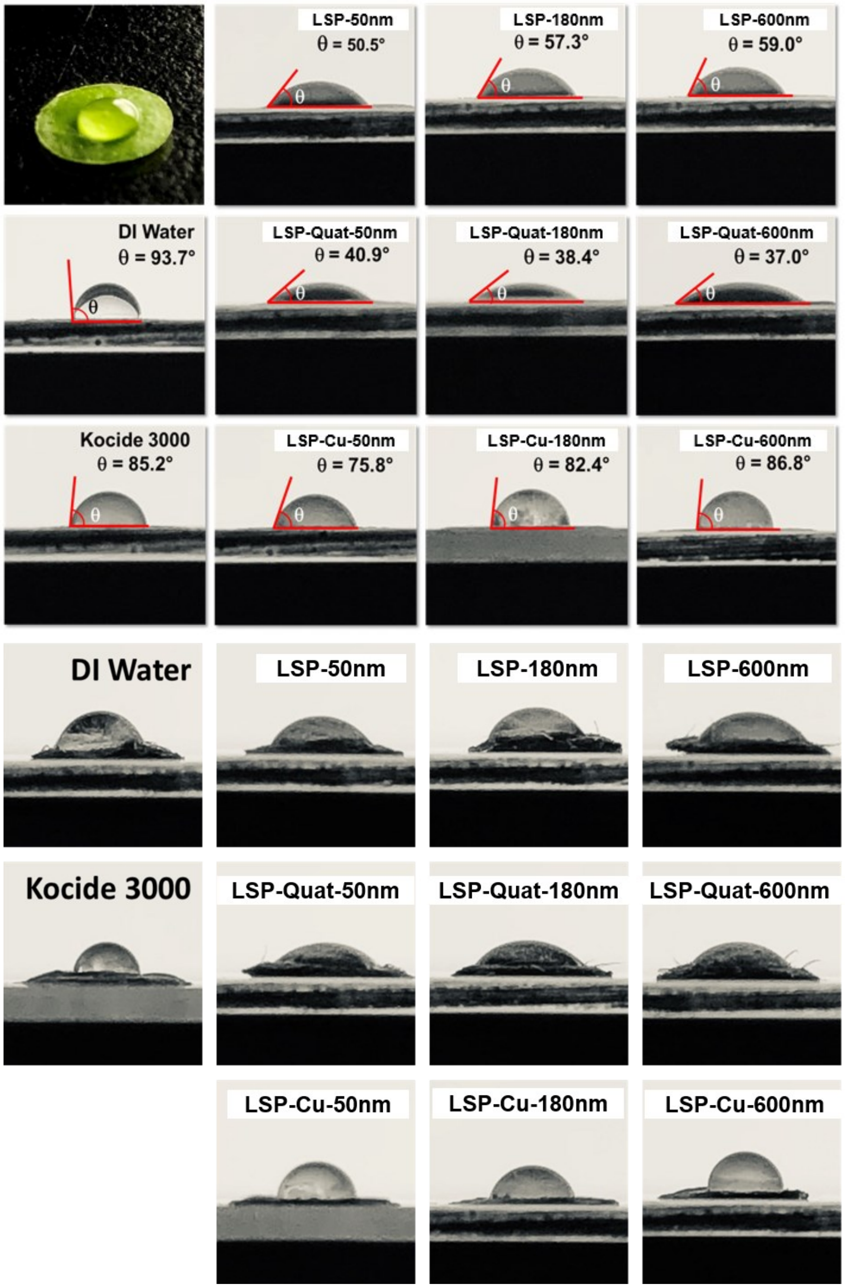

Figure S4: Leaf surface wetting measurements (contact angle) on young citrus leaf surface (top panel) and tomato leaves (bottom panel) for water, Kocide 3000, LSP-50nm, LSP-180nm, LSP-600nm, LSP-Cu-50nm, LSP-Cu-180nm, LSP-Cu-600nm, LSP-Quat-50nm, LSP-Quat-180nm, and LSP-Quat-600nm. Qualitative improvements of surface wetting could be observed for LSP particles and LSP-Quat compared to LSP- Cu, DI water, and Kocide 3000. Contact angle values could not be determined accurately due to the variability from leaf to leaf together with high roughness, and fragility of the tomato leaves.

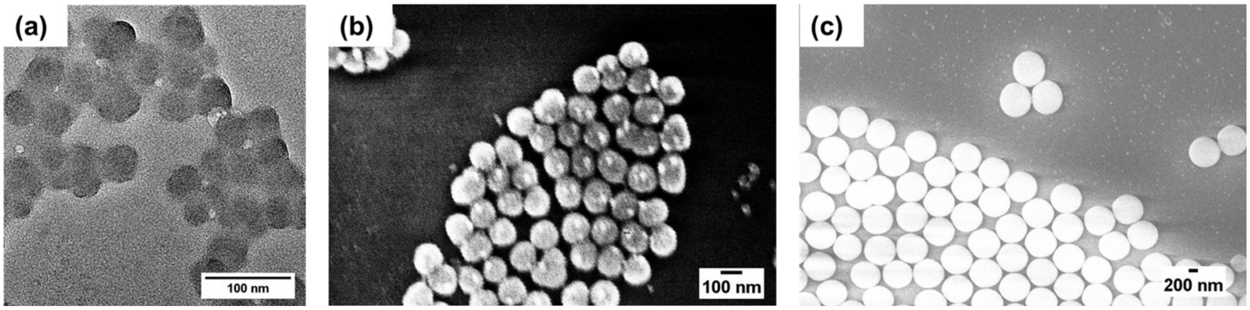

Figure S5: Electron microscopy images of silica seeds. (a) TEM image of seed #1, particle size 30 - 45 nm, (b) and (c) are SEM images of seed #2 and seed # 3, particle sizes 120 to 150 nm and 550 to 600 nm, respectively.

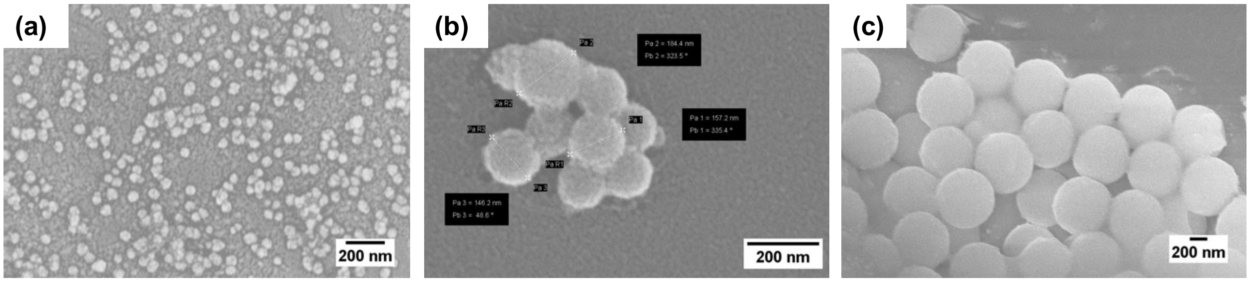

Figure S6: SEM images of LSP particles. Size of particles were found to (a) LS-50nm, particles between 40 to 60 nm, (b) LSP-180nm, particles between 140 to 190 nm. (c) LSP-600nm particles, particles between 570 to 650 nm.

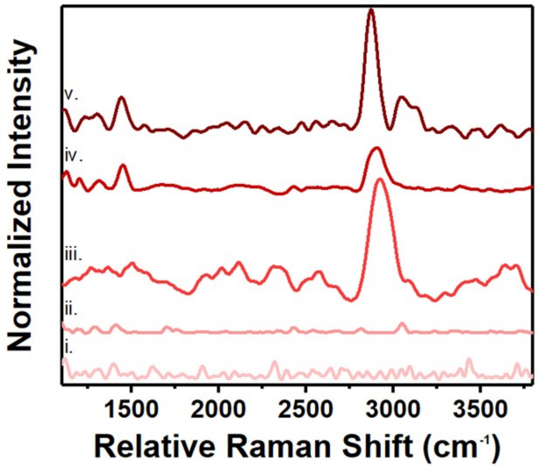

Figure S7: Raman spectral analysis of LSP chemical components. i. $CuSO_4$, ii. LSP-Cu-50nm, iii. LSP-Quat-50nm, iv. Inert silica core-shell NPs (seed#1), v. LSP-50nm.

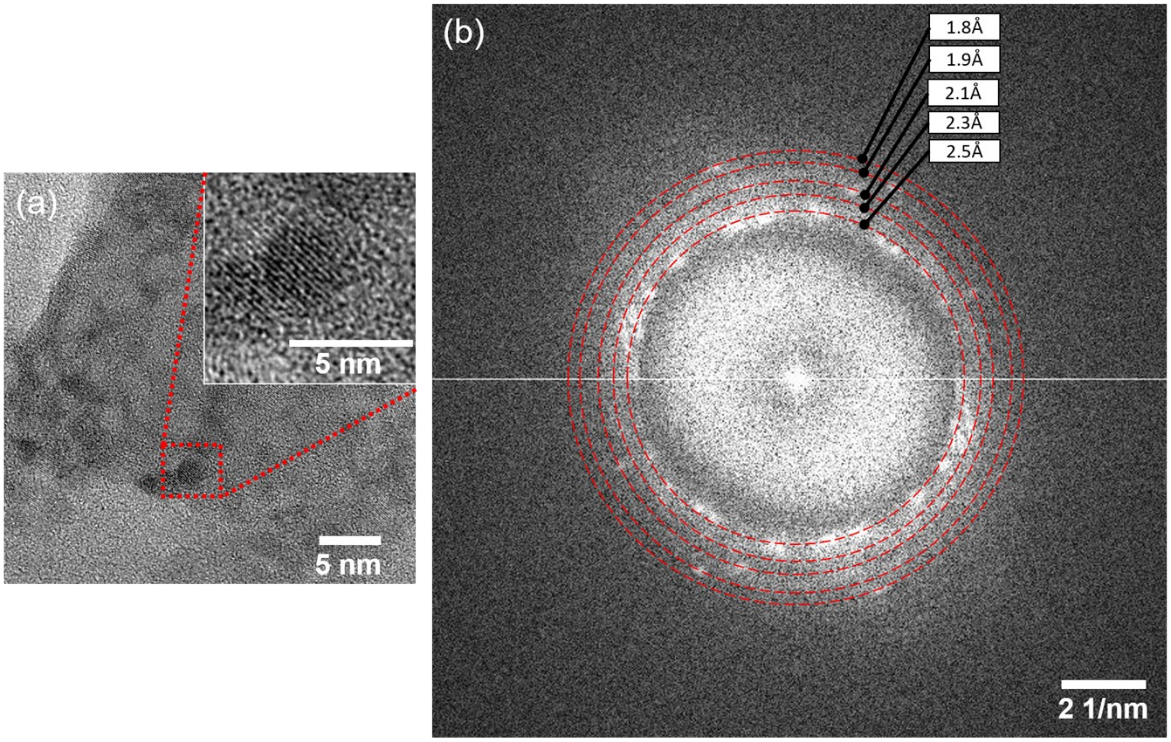

Figure S8: Large view of the TEM FFT of Cu Cluster in Fig.1(f) with markers for the d-spacing indicated in Table S1.

Table S1: d-spacing values calculated for Cu NPs shown above in the LSP particle shell, compared to copper hydroxide crystal (JCPDS: #13-0420)

[h k l]	Calculated d-spacing values for Cu nanoclusters in LSP shell (Å)	d-spacing values for Cu hydroxide (JCPDS # 13-0420) (Å)
[1 1 1]	2.501	2.500
[1 3 0]	2.265	2.263
[1 3 1]	2.090	2.078
[1 1 2]	1.932	1.929
[0 6 0]	1.780	1.767

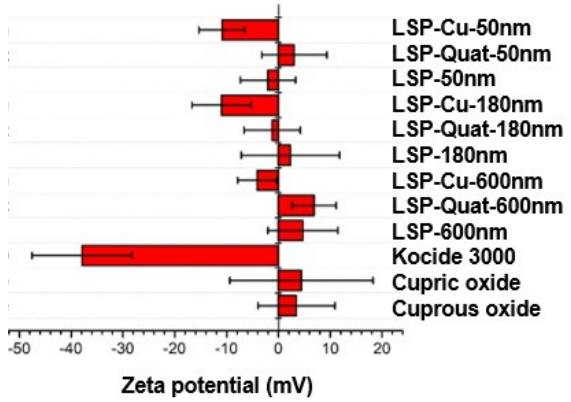

Figure S9: Zeta potential of the particles in LSP formulations and control solutions. Kocide 3000 and LSP-Cu formulations all present negative Zeta potentials. The presence of Quat in the formulation leads to an increase in Zeta potentials, to nearly zero or slightly average positive values.

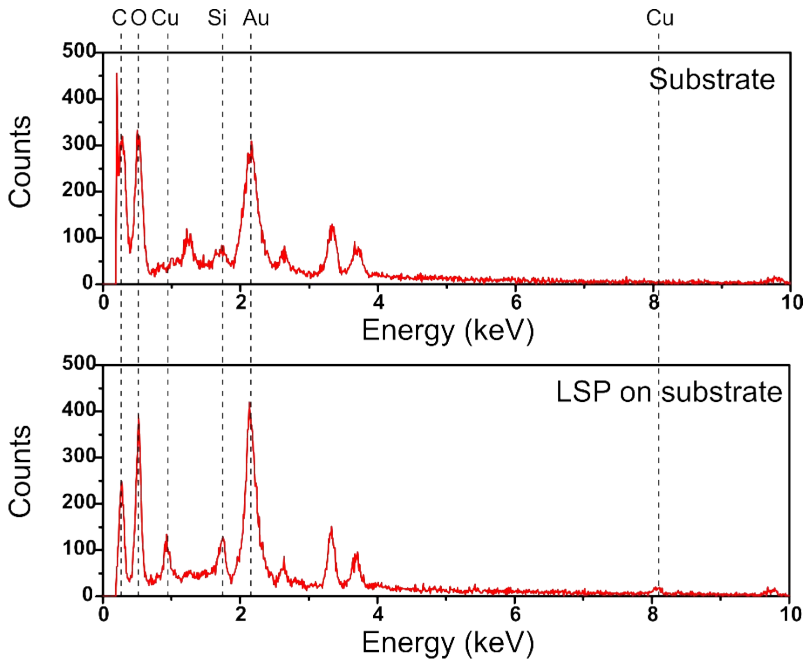

Figure S10: Elemental analysis of LSP-50nm performed using Noran system 7 energy-dispersive x-ray fluorescence spectroscope (EDS) using a 15 kV and a working distance of 15 mm.
